# A comparison of four methods for detecting weak genetic structure from marker data

**DOI:** 10.1002/ece3.237

**Published:** 2012-05

**Authors:** Owen R Jones, Jinliang Wang

**Affiliations:** 1 Institute of Zoology, Zoological Society of LondonRegents Park, London, NW1 4RY; 2 Max Planck Institute for Demographic ResearchKonrad Zuse Str. 1, D-18057, Rostock, Germany

**Keywords:** Conservation genetics, dispersal, gene flow, genetic structure, population genetics

## Abstract

Genetic structure is ubiquitous in wild populations and is the result of the processes of natural selection, genetic drift, mutation, and gene flow. Genetic drift and divergent selection promotes the generation of genetic structure, while gene flow homogenizes the subpopulations. The ability to detect genetic structure from marker data diminishes rapidly with a decreasing level of differentiation among subpopulations. Weak genetic structure may be unimportant over evolutionary time scales but could have important implications in ecology and conservation biology. In this paper we examine methods for detecting and quantifying weak genetic structures using simulated data. We simulated populations consisting of two putative subpopulations evolving for up to 50 generations with varying degrees of gene flow (migration), and varying amounts of information (allelic diversity). There are a number of techniques available to detect and quantify genetic structure but here we concentrate on four methods: *F*_ST_, population assignment, relatedness, and sibship assignment. Under the simple mating system simulated here, the four methods produce qualitatively similar results. However, the assignment method performed relatively poorly when genetic structure was weak and we therefore caution against using this method when the analytical aim is to detect fine-scale patterns. Further work should examine situations with different mating systems, for example where a few individuals dominate reproductive output of the population. This study will help workers to design their experiments (e.g., sample sizes of markers and individuals), and to decide which methods are likely to be most appropriate for their particular data.

## Introduction

The genetic structuring of populations, a systematic variation in allele frequencies through space, is common in wild populations and is primarily the result of the opposing forces of genetic drift and gene flow, and spatial variation in natural selection. Genetic drift is the change in allele frequencies through time caused by the random sampling of parental alleles, along with the role of chance in governing the survival to reproduction of the offspring. This process becomes particularly important when population size is small. Thus, through time, allele frequencies in a population can change by chance alone. Gene flow works to homogenize spatial genetic variation by moving alleles through space (Slatkin 1987). Barriers to gene flow can be physical, such as rivers or mountain ranges, which prevent individuals or gametes moving between regions, or related to demographic rates (such as hunting or predation pressure). There are also the natural limits imposed by the organism's dispersal ability, which can be especially restricted in plants and small animals. In some cases, gene flow between regions can be reduced to zero, but often gene flow is merely reduced by some degree. When gene flow is reduced, the opportunity for divergence in allele frequencies via genetic drift is enhanced. Therefore, so is the development of genetic structure. In addition, with time, any variation in the success of particular gene variants due to regional differences in natural selection will generate spatial variation in allele frequencies.

The fact that population genetic structure arises from these distinct phenomena means that observations of spatial variation in allele frequencies can be used to infer the processes that gave rise to them. Consequently, using genetic data alone, it is possible to make inferences about gene flow and therefore dispersal behavior. Following this, inferences can be made about the mating system, social and phylogeographic structure, and population dynamics of the population (Nutt 2008). These inferences provide vital information for the effective management of endangered species.

There are numerous approaches to estimating gene flow indirectly using genetic data. One method is to regress measures of genetic distance, such as *F*_ST_ (e.g., Rousset 1997, 2000) or relatedness (Hardy and Vekemans 1999), against the distance between individuals. Alternatively, even nonspatially explicit data can be revealing. For example, a measure of relatedness can be informative, because dispersal is likely to influence local relatedness patterns (Mossman and Waser 1999; Banks et al. 2002; Hazlitt et al. 2004): as dispersal increases, relatedness at a particular sampling location will tend to decrease.

Besides their utility in revealing current dispersal patterns, genetic structure information can also be used to elucidate phylogeographic history. For example, population fragmentation to isolated refugia during glacial periods would result in divergence of allele frequencies due to genetic drift and selection. In addition, as populations expand into newly available habitats differentiation and divergence can occur via selection and hitchhiking and, if further immigration is limited, drift (Hewitt 1996; Johnson et al. 2007). In some cases, such events may be regarded as founder events and lead to founder effects (a special case of drift). Information on gene flow and population structure is potentially very valuable for conservation as this information could provide early warning of changing distribution patterns, for example due to climate change.

There are several methods in use for detecting and assessing genetic structure. We briefly discuss four of them because they will be compared in this simulation study.

The first method makes use of one of Wright's *F*-statistics, *F*_ST_ (Wright 1965). *F*_ST_ is a widely used statistic in population genetics. It is an estimate of the proportion of genetic diversity among populations: when *F*_ST_ is zero, there is no differentiation; and when it is 1, the populations are fixed for different alleles (Hartl and Clark 1997). We would thus expect *F*_ST_ to decline as gene flow increases, for example via increased dispersal ability or declining distance between subpopulations. One criticism of *F*_ST_ for inferring gene flow is that the underlying model assumes that the population is at an equilibrium state, and therefore, the method may not be suitable to studies on short timescales (Whitlock and McCauley 1999; Paetkau et al. 2004).

The second method uses individual-based population assignment tests to estimate structure. With this method, the likelihood of an individual originating from each of a number of candidate subpopulations is calculated using the individual multilocus genotype and the allele frequencies of the candidate subpopulations, and the individual is assigned to the subpopulation that has the maximum likelihood. This assignment method was described in Paetkau et al. (2004) and was called the frequency method in Cornuet et al. (1999). Where the subpopulations in which the individuals are found correlate strongly with the subpopulations to which they are assigned, the population may be considered structured. On the other hand, where the pattern of assignment appears to be random, the population is considered unstructured.

The third method makes use of pairwise relatedness information (i.e., between pairs of individuals in the population; Wang 2002). Generally, as migration increases, pairwise relatedness within subpopulations will tend to decrease relative to that between subpopulations. By comparing the average relatedness within and between subpopulations, it is possible to infer population structure.

The fourth and final method of detecting and quantifying genetic structure has received little attention in the past and makes use of kin structure, or inferred sibship, information in the population. Where the population is unstructured, sibships (and indeed any relationship) will be distributed randomly amongst subpopulations. However, when the population is structured, sibships are more likely to be found within subpopulations rather than between subpopulations (Piyapong et al. 2011).

Among the four methods, the last two are similar but with some important differences. The relatedness method estimates a continuous quantity between 0 and 1 measuring the proportion of alleles that are identical by descent shared between individuals, while the sibship method infers a discrete genealogical relationship, sibship, against the unrelated. The former is more general and does not require any specific sample structure, while the latter assumes a single-generation sample of individuals taken from a population, so that, close across-generation relationships (e.g., parent–offspring) are impossible. However, relatedness is usually much more difficult to estimate accurately than relationship (sibship), especially for a single-generation sample in which only a few well-differentiated candidate relationships are available (Wang 2006).

In this paper, we assess the efficacy of these four methods for detecting weak genetic structure under a range of conditions using simulated data. We vary the amount of genetic information in the data (in the form of number allelic diversity), and the length of time allowed for genetic structure to develop (and therefore strength of genetic structure). Our results will help biologists who are considering analytical methods for their study system.

## Materials and Methods

Because it is analytically intractable to compare the accuracy and power of the four methods, we used simulations instead. We simulated the evolution of a pair of subpopulations split from the same ancestral population with different migratory characteristics. By varying the number of generations allowed to have elapsed since the subpopulations were separated, and the amount of migration between subpopulations, we could control the amount of genetic structuring that would result.

### Simulating populations

We first randomly generated a set of multilocus genotypes for 500 diploid individuals in a single large population, given the allele frequencies generated from a uniform Dirichlet distribution and Hardy–Weinberg and linkage equilibria. We then randomly assigned half (250) of the individuals from this initial population to each of two separate subpopulations (A and B). Each subpopulation has discrete generations and evolves under a monoecious mating system. In this system, 250 pairs of individuals were randomly selected (with replacement) and allowed to mate and produce a single offspring to contribute to the next generation. Population size thus remained constant through time. At each generation, a certain amount of migration between the subpopulations was allowed to control the rate of development of population structure. This migration was stochastic, with each individual having a certain probability of migrating to the other subpopulation. In addition, we allowed a small mutation rate, according to the *k*-allele model, of *u*= 0.0001 (where *k* is the number of alleles per locus). For this model, at each locus, we drew a random number (*m*) from a Poisson distribution with a mean of 500*u* to determine the number of allelic copies that experience a mutation at that locus in a subpopulation. Then *m* allelic copies were then selected at random from the population and each mutating allele copy in question was replaced by one of the *k* alleles (including the existing allele) chosen at random with an equal probability of 1/*k*. We validated this simulation procedure by comparing the *F*_ST_ values calculated from simulated data to theoretical expectations (see Supporting Information).

Under the simulation model, therefore, the genetic structure of the population can increase from nonexistent at *t*= 0 to substantial as time progresses and the allele frequencies of the subpopulations diverge via genetic drift. The development of genetic structure will be constrained by migration of individuals between subpopulations, and will also be influenced by mutations. Therefore, the rate of development of genetic structure will be fastest when the migration rate is zero, and the rate will decline as the rate of migration increases.

We established an experimental design with migration rate, and degree of polymorphism, varying as follows. Migration was defined as the probability of individuals in the population that moved between subpopulations. It was either zero, negligible (0.04, which corresponds to, on average, one individual per generation in our simulations), small (0.10) or high (0.20); number of loci was fixed at 10; and finally, polymorphism, defined as the number of alleles per locus, was low (5), medium (10), or high (20). For each case, we conducted 50 replicate simulations. We used R (R Development Core Team 2009) to conduct all of these simulations.

### Estimating genetic structure

We tested the utility of four approaches for detecting population genetic structures. In each case, we used the marker data from a randomly selected subset of the populations (20% of the individuals, i.e., 50 from each subpopulation) rather than the entire population in inferring population structures. We did this to mimic incomplete sampling of the population, which is the norm in most studies. Individuals were sampled after the migration events.

#### F_ST_ method

We estimated *F*_ST_ using the *fstat* function in the R package “*adegenet*” (Jombart 2008). Significance of the observed *F*_ST_ value was determined by a permutation test with 500 permutations. For each replicate of the permutation test, the location of the individuals in the population (i.e., subpopulation *A* or subpopulation *B*) was randomly permuted, and *F*_ST_ recalculated. The *P*-value was then taken to be the proportion of the 500 resampled *F*_ST_ values that were greater than the observed value. Thus, we regard the population as significantly structured when only 25 or less of the 500 (5%) permutations produce *F*_ST_ values greater than or equal to the observed *F*_ST_ value.

#### Population assignment method

The population assignment method examines each individual in turn and, based on its genotypes and the subpopulation allele frequencies, assigns it to one of the putative subpopulations that has the maximum likelihood. A popular program for doing this is STRUCTURE (Pritchard et al. 2000), but we use our own algorithm programmed in R. Like STRUCTURE, our algorithm estimates the likelihood of assignment of each individual to each subpopulation based on Hardy–Weinberg and linkage. If the proportion of “correct” individual assignments (i.e., individuals are assigned to the subpopulation from which they are sampled) is significantly higher than that of “misassignments” (i.e., individuals are not assigned to the subpopulation from which they are sampled), then a genetic structure of the population is detected. Otherwise, the population is regarded as genetically unstructured. As above, we estimated significance for this method using a permutation test with 500 permutations. Again we permuted the population assignments (i.e., subpopulation *A* or subpopulation *B*) and calculated the proportion that were misassigned. The *P*-value for the significance of population structure was taken to be the proportion of the 500 permutations where the misassignment proportion in the permuted set was lower than the empirical observation.

#### Relatedness method

We use COANCESTRY (Wang 2010) to estimate pairwise relatedness using five different moment estimators: Wang's estimator (Wang 2002), an estimator described by both Lynch (1998) and Li et al. (1993), Lynch and Ritland's (1999) estimator, Ritland's (1996) estimator, and Queller and Goodnight's (1989) estimator. We calculated the average within-population relatedness for dyads in which both individuals come from the same subpopulation, and the average between-population relatedness for dyads in which individuals come from different subpopulations. A significant difference between the average within-population relatedness and the average between-population relatedness indicates the genetic structure. We determined the significance of genetic structure using a permutation test with 500 permutations. With this approach, we took the *P*-value to be the proportion of randomized between-population relatedness estimates that were greater than the observed between-population relatedness estimate.

#### Sibship method

We used COLONY (Wang 2004; Jones and Wang 2010) to reconstruct sibships of the sampled individuals and to investigate the distribution of sibship within and between subpopulations. The idea behind this sibship method is that, with a completely mixed pair of subpopulations (i.e., no structure), the proportion of sibdyads that occur within the same subpopulation (i.e., *A*–*A* or *B*–*B*) and the proportion that occur between the two subpopulations (i.e., *A*–*B* or *B*–*A*) will be similar. As the population becomes more structured due to less migration, the probability of an individual from population *A* having siblings in population *B* and vice versa would decrease. This method is expected to be especially useful in the case of weak structure due to a high migration rate, where *F*_ST_ and other methods have little power.

Once we identified the numbers of sibships that occurred within- and between-subpopulations we used a permutation test to determine whether this was significantly different from the null expectation that the sibship distributions were simply random with respect to subpopulation identity. We did this by permuting (500 times) the identity of the subpopulation from which the individuals were sampled and recalculating the number of sibships within- and between-subpopulations. Using this approach, we took the *P*-value to be the proportion of the resampled within-subpopulation sibship frequency estimates that were greater than the observed within-subpopulation sibship frequency.

## Results

**Figure 1:**
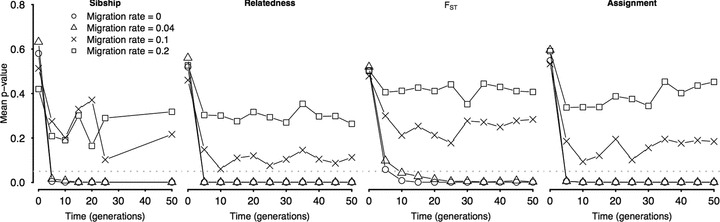
The effect of increasing levels of migration on the significance (*P*-values) of tests for genetic structure using four different methods. The numbers of loci and alleles were both fixed at 10. The symbols indicate the probability of migration of individuals in the simulations (0 = circles, 0.04 = triangles, 0.1 = crosses, 0.2 = squares). Each point represents the mean of *P*-values across replicates.

All four methods produce qualitatively similar results. They all show that as structure develops, for example, with an increasing number of generations, the significance of the tests for genetic structure increases (i.e., the *P*-value of the tests tend to decline). When migration rate (i.e., gene flow) between the subpopulations is low, this decline in *P*-value (i.e., an increase in the significance of genetic structuring) is very rapid, but when migration rates are high, significant structuring does not occur (Fig. 1). The five relatedness measures that we estimated produce almost identical results, and we therefore, only present one of them, Wang's (2002) estimator.

**Figure 2:**
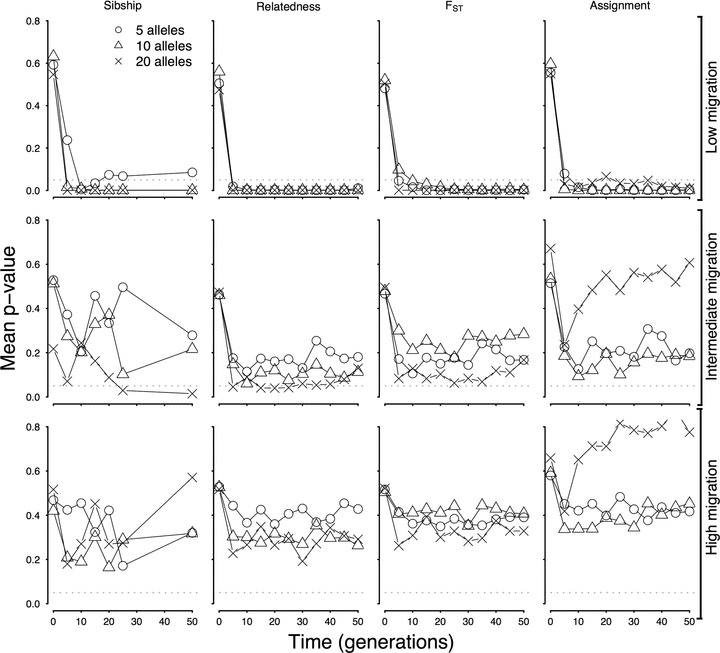
The effect of the number of alleles on significance of tests for genetic structure using four different methods, under three different migration conditions. The top row shows data from simulations with low levels of migration (probability of migration of 0.04); the middle and bottom rows show data from simulations where individuals have probabilities of migration of 0.1 and 0.2, respectively. The symbols indicate indicates the number of alleles (5 = circles, 10 = triangles, 20 = crosses) at each of 10 loci.

Furthermore, increasing the amount of information in the dataset by increasing allelic diversity tends to increase the power, and therefore, the significance of the test results (Fig. 2).

**Figure 3:**
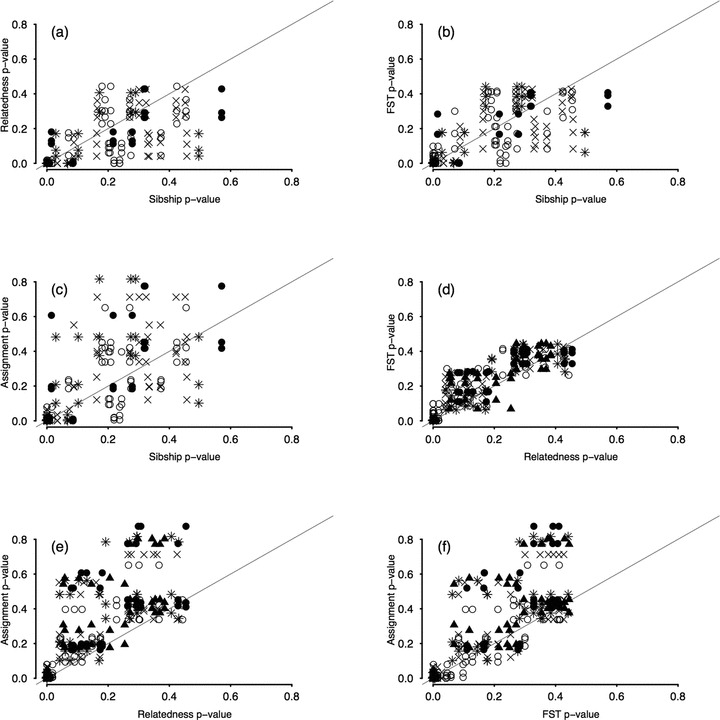
The association between the *P*-values produced by the four methods. The symbols used for the points indicate the number of generations that have elapsed (1–10 = open circles, 11–20 = crosses, 21–30 = asterisks, 31–40 = filled triangles, 41–50 = filled circles). The plot includes points from simulations with 5, 10, and 20 alleles.

The correlation between the *P*-values of the four methods was high, with Pearson's correlation coefficients ranging from 0.76 to 0.94. Nevertheless, it was apparent that the assignment method tended to perform consistently poorly compared to the other three methods (Fig. 3). If pairs of methods are equally effective, then the points in Figure 3 should fall close to the 1:1 line, because the *P*-values would tend to be similar. If one method performs relatively poorly, then the *P*-values of the poor performer would tend to be higher than those of the better performer, and the points would fall away from the 1:1 line. This is the case for the assignment test where the *P*-values of the tests for significant structure tended to be higher than those produced by the other three methods (Fig. 3).

## Discussion

Our results show that all four of our selected methods for detecting genetic structure behave in a qualitatively similar way: as time elapses, and the number of generations increases, the statistical significance of the population structure also increases (i.e., the *P*-value decreases). It is therefore apparent that the method chosen to detect structure is not necessarily particularly important if the scale of the study is fairly broad and fine-scale patterns are not considered.

However, based on its obtained levels of statistical significance compared to the other three methods, the assignment method performed relatively poorly. This finding is similar to Nutt's (2008) analysis, which found that assignment testing was of limited use in detecting fine-scale dispersal patterns. Therefore, based on these and our current findings, we recommend that the assignment method should be avoided when attempting to detect finer-scale patterns. We note, however, that Lee (2007) found that assignment methods tended to outperform the *F*_ST_-based methods for their dataset. Their conclusion is not generalized because it is based on a single dataset. In our simulations, it is also possible that assignment methods outperform the *F*_ST_-based method for a particular replicate dataset, although on an average they perform poorly. Another important factor is the computing time involved in conducting these analyses. The times for most of these methods is trivial, however, computation times for the sibship analysis can be relatively long, and unpredictable. This should not be much of a hindrance for most applications, but when repeated analyses as in a simulation study are required it may become problematic.

Our simulations captured a simple situation, where every individual in the population is equally likely to produce offspring. These circumstances are rather unlikely in wild animals and our results must be interpreted with a degree of caution because other mating characteristics could produce different results. For example, where mating is nonrandomly distributed across the population, and a small number of individuals dominate reproduction, sibships will be large. In such a case, we would expect the sibship method to outperform the other methods.

Furthermore, allele frequencies for each population were calculated from the genotypes of individuals sampled from within the population, and may thus include immigrants. Therefore, allele frequencies are potentially misspecified when migration causes a change in the allele frequencies from one generation to another. Therefore, any method that relies heavily on an estimate of allele frequencies, such as the assignment method, may be inaccurate when gene flow is high. In addition, using highly polymorphic markers with a greater number of alleles may not necessarily improve the accuracy of the analysis because, as the number of alleles increases, allele frequencies become more sensitive to gene flow. This notion is supported by Figure 2, which shows that the assignment method becomes less powerful with more alleles per marker when the migration rate is high. It is technically possible to use the multilocus genotype information to identify and remove immigrants from the analysis (Rannala and Mountain 1997). This procedure would improve the accuracy of allele frequency estimates and therefore the estimation of genetic structure. The degree of improvement that can be obtained with this approach deserves further investigation.

## Supporting Information - Evolutionary Analysis

**Figure S1.** Comparison of *F*_ST_ derived from our simulated data from a two population system with a fixed migration rate, and with those expected from theoretical predictions. The red curves represent the theoretical predictions, and the green points/lines represent the outcome of our simulations. The three different curves (from the top to the bottom) represent three different levels of migration rate 0, 0.0025, and 0.01.

## References

[b1] Banks S, Skerrat L, Taylor A (2002). Female dispersal and relatedness structure in common wombats (*Vombatus ursinus*. J. Zool.

[b2] Cornuet J-M, Piry S, Luikart G, Estoup A, Solignac M (1999). New methods employing multilocus genotypes to select or exclude populations as origins of individuals. Genetics.

[b3] Hardy OJ, Vekemans X (1999). Isolation by distance in a continuous population: reconciliation between spatial autocorrelation analysis and population genetics models. Heredity.

[b4] Hartl DL, Clark AG (1997). Principles of population genetics.

[b5] Hazlitt SL, Eldridge MDB, Goldizen AW (2004). Fine-scale spatial genetic correlation analyses reveal strong female philopatry within a brush-tailed rock-wallaby colony in southeast Queensland. Mol. Ecol.

[b6] Hewitt GM (1996). Some genetic consequences of ice ages, and their role in divergence and speciation. Biol. J. Linn. Soc.

[b7] Johnson JA, Burnham KK, Burnham WA, Mindell DP (2007). Genetic structure among continental and island populations of gyrfalcons. Mol. Ecol.

[b8] Jombart T (2008). adegenet: a R package for the multivariate analysis of genetic markers. Bioinformatics.

[b9] Jones OR, Wang J (2010). COLONY: a program for parentage and sibship inference from multilocus genotype data. Mol. Ecol. Resour.

[b10] Lee PLM, Luschi P, Hays GC (2007). Detecting female precise natal philopatry in green turtles using assignment methods. Mol. Ecol.

[b11] Li C, Weeks D, Chakravarti A (1993). Similarity of DNA fingerprints due to chance and relatedness. Hum. Hered.

[b43] Lynch M (1988). Estimation of relatedness by DNA fingerprinting. Mol. Biol. Evol.

[b13] Lynch M, Ritland K (1999). Estimation of pairwise relatedness with molecular markers. Genetics.

[b14] Mossman CA, Waser PM (1999). Genetic detection of sex-biased dispersal. Mol. Ecol.

[b15] Nutt KJ (2008). A comparison of techniques for assessing dispersal behaviour in gundis: revealing dispersal patterns in the absence of observed dispersal behaviour. Mol. Ecol.

[b16] Paetkau D, Slade R, Burden M, Estoup A (2004). Genetic assignment methods for the direct, real-time estimation of migration rate: a simulation-based exploration of accuracy and power. Mol. Ecol.

[b17] Piyapong C, Butlin RK, Faria JJ, Scruton KJ, Wang J, Krause J (2011). Kin assortment in juvenile shoals in wild guppy populations. Heredity.

[b18] Pritchard JK, Stephens M, Donnelly P (2000). Inference of population structure using multilocus genotype data. Genetics.

[b19] Queller DC, Goodnight KF (1989). Estimating relatedness using genetic markers. Evolution.

[b20] R Development Core Team (2009). R: a language and environment for statistical computing.

[b21] Rannala B, Mountain JL (1997). Detecting immigration by using multilocus genotypes. Proc. Natl. Acad. Sci. U.S.A.

[b22] Ritland K (1996). Estimators for pairwise relatedness and individual inbreeding coefficients. Genet. Res.

[b23] Rousset F (1997). Genetic differentiation and estimation of gene flow from F-statistics under isolation by distance. Genetics.

[b24] Rousset F (2000). Genetic differentiation between individuals. J. Evol. Biol.

[b25] Slatkin M (1987). Gene flow and the geographic structure of natural populations. Science.

[b26] Wang J (2002). An estimator for pairwise relatedness using molecular markers. Genetics.

[b27] Wang J (2004). Estimating pairwise relatedness from dominant genetic markers. Mol. Ecol.

[b28] Wang J (2006). Informativeness of genetic markers for pairwise relationship and relatedness inference. Theor. Popl. Biol.

[b60] Wang J (2011). COANCESTRY: a program for simulating, estimating and analysing relatedness and inbreeding coefficients. Mol. Ecol. Res.

[b30] Whitlock MC, McCauley DE (1999). Measures of gene flow and migration: F_ST_ ≠ 1/(4Nm+1). Heredity.

[b31] Wright S (1965). The interpretation of population structure by F-statistics with special regard to systems of mating. Evolution.

