# Supplementary material for: A comparison of four methods for detecting weak genetic structure from marker data
Source: Ecol Evol. 2012 May;2(5):1048–55. doi: 10.1002/ece3.237 (PMC3399169; doi:10.1002/ece3.237)
Supplement: Supplementary file 1 [file ece30002-1048-SD1.doc]

Supplemental Material for Jones, O.R. and Wang, J. A comparison of four methods for detecting weak genetic structure from marker data. Ecology and Evolution.

**Appendix S1: Validation of our simulations with theoretical predictions.**

Figure S1: Comparison of F_ST_ derived from our simulated data from a two population system with a fixed migration rate, and with those expected from theoretical predictions. The red curves represent the theoretical predictions, and the green points/lines represent the outcome of our simulations. The 3 different curves (from the top to the bottom) represent 3 different levels of migration rate 0, 0.0025 and 0.01.


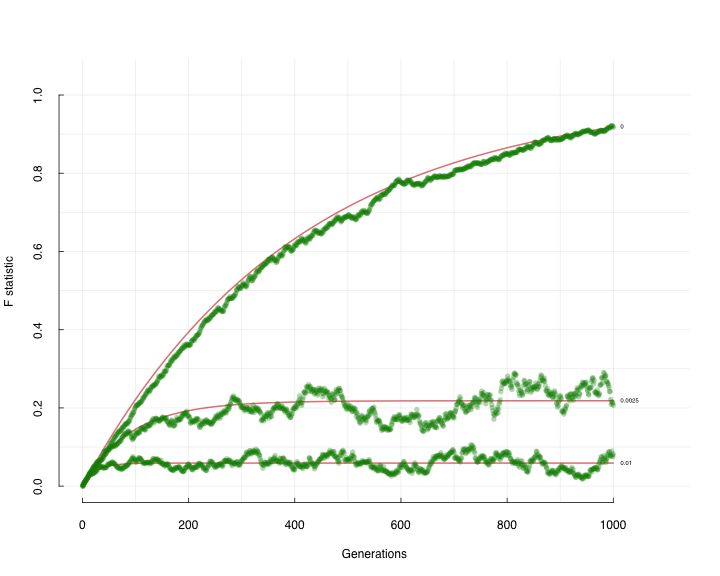


**The theoretical predictions were calculated as follows:**

In our system with *N* individuals divided among two subpopulations, the parameters of interest are the inbreeding coefficient (*f*), the coancestry coefficient between a pair of individuals within a subpopulation (*g*), and the coancestry coefficient between a pair of individuals, with one from one subpopulation and one from the other.

Initially, at generation 0, we have *f_0_*=*g_0_*=*h_0_*=0. Then, at generation t+1 (before migration) we have, for *f**, *g**, and *h** (the asterisk signifies that the measures are before migration):


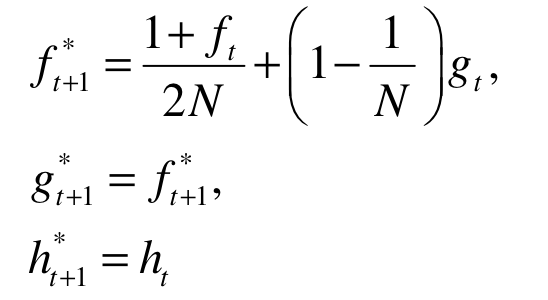


Subsequently, the recurrence equations to calculate *f_t+1_*, *g_t+1_* and *h_t+1_*, assuming a migration rate of *m,* are:


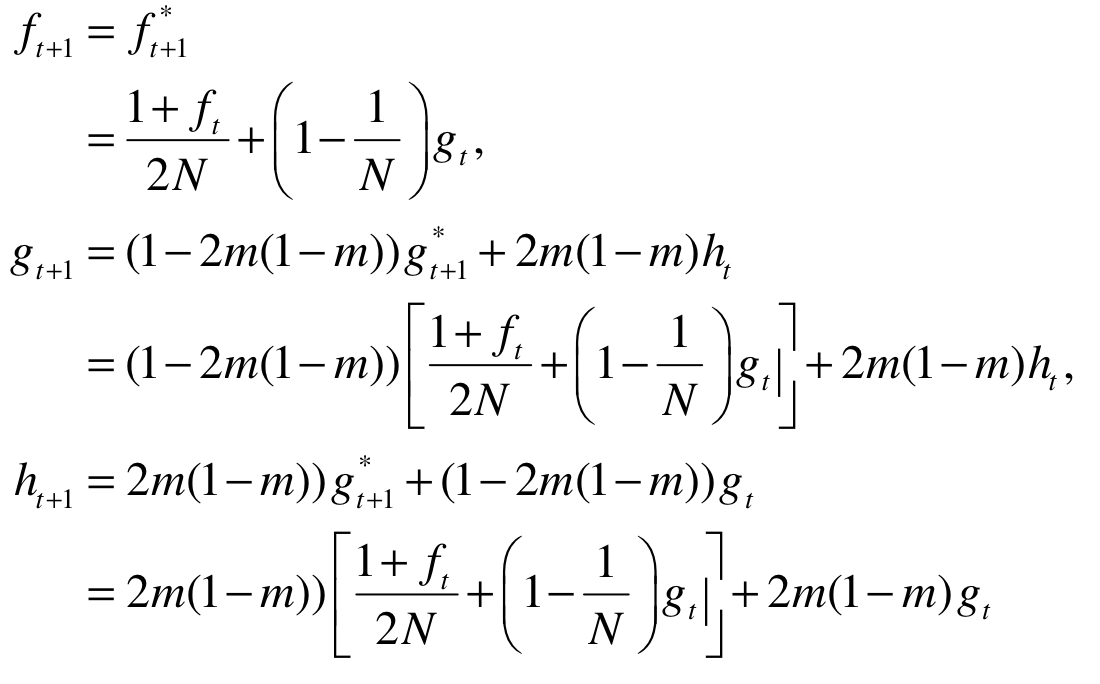


From the initial conditions, and the recurrence equations (above), we can obtain the values for *f*, *g* and *h* for any generation *t*, given the parameters *m* and *N*.
